# Experimental evidence of mechanical lumpy skin disease virus transmission by *Stomoxys calcitrans* biting flies and *Haematopota spp*. horseflies

**DOI:** 10.1038/s41598-019-56605-6

**Published:** 2019-12-27

**Authors:** C. Sohier, A. Haegeman, L. Mostin, I. De Leeuw, W. Van Campe, A. De Vleeschauwer, E. S. M. Tuppurainen, T. van den Berg, N. De Regge, K. De Clercq

**Affiliations:** 1 Sciensano, Groeselenberg 99, 1180 Brussel, Belgium; 2 Independent Veterinary Consultant for Lumpy skin disease, Sheeppox and Goatpox, Helsinki, Finland

**Keywords:** Entomology, Skin diseases

## Abstract

Lumpy skin disease (LSD) is a devastating disease of cattle characterized by fever, nodules on the skin, lymphadenopathy and milk drop. Several haematophagous arthropod species like dipterans and ticks are suspected to play a role in the transmission of LSDV. Few conclusive data are however available on the importance of biting flies and horseflies as potential vectors in LSDV transmission. Therefore an *in vivo* transmission study was carried out to investigate possible LSDV transmission by *Stomoxys calcitrans* biting flies and *Haematopota spp*. horseflies from experimentally infected viraemic donor bulls to acceptor bulls. LSDV transmission by *Stomoxys calcitrans* was evidenced in 3 independent experiments, LSDV transmission by *Haematopota spp*. was shown in one experiment. Evidence of LSD was supported by induction of nodules and virus detection in the blood of acceptor animals. Our results are supportive for a mechanical transmission of the virus by these vectors.

## Introduction

Lumpy skin disease (LSD) is a viral disease of cattle caused by lumpy skin disease virus (LSDV). LSDV is one of the most important animal poxviruses because of the serious economic consequences in cattle. The World Organization for Animal Health (OIE) categorizes LSD as a notifiable disease^1^. It is characterized by fever, reduced milk production and skin nodules. Mastitis, swelling of peripheral lymph nodes, loss of appetite, increased nasal discharge and watery eyes are also common. Temporary or permanent infertility occur among infected cows and bulls. The disease can cause high morbidity and low mortality^2–4^.

LSDV is endemic in southern, central, eastern and western Africa. Before 2012, only scarce LSDV outbreaks were reported in the Middle East region. However, currently, LDS spread to most African and Middle East countries, and recently it affects eastern and south-eastern European countries (Balkan countries)^5,6^.

Our ability to devise effective, safe and economically sound LSD control programs is greatly hampered by key gaps in our understanding of the disease. An important gap is the means by which the virus is transmitted from animal-to-animal. In particular the role of vector transmission and the vectors involved is unclear^7^. Almost all hematophagous dipterans (stable flies, horseflies, mosquitoes) and ticks were already suggested to be potential vectors in the transmission of LSDV between cattle.

Experimentally, female *Aedes aegypti* mosquitoes have been shown to transmit LSDV mechanically from infected to susceptible cattle^8^. The potential role of ixodid ticks (*Amblyomma hebraeum*, *Rhipicephalus appendiculatus*, *Rhipicephalus decoloratus*) in mechanical and intrastadial transmission of LSDV has also been demonstrated^9–13^. Attempts to attain potential transmission of LSDV by the *Anopheles stephensi* mosquito and *Culicoides nubeculosus* biting midges were not successful^14^, although the presence of LSDV DNA was demonstrated in Culicoides during the recent LSD epidemic in Turkey^15^.

No conclusive data are available on the possible LSDV transmission by the stable fly *S. calcitrans* and by Tabanidae horseflies. Both insects are considered as mechanical vectors of viral diseases and are abundantly present in Belgium^16,17^. Stable flies have shown to be able to mechanically transmit capripoxvirus between sheep^18^, and live LSDV has been isolated from *Stomoxys calcitrans* after feeding on infected cattle^19^. Although epidemiological observations already showed a high relative abundance of *S. calcitrans* with the occurrence of LSD on dairy farms^20,21^, LSDV transmission by *S. calcitrans* could not be demonstrated in a previous experimentally study^14^. Probably, the 24 h time period between feeding *S. calcitrans* on an infectious host and afterwards on a susceptible host was too long to allow mechanical transmission since the pathogen lost its infectiousness and/or was removed from the mouth parts within this time period. No data on LSDV transmission by Tabanidae are already available.

We therefore decided to focus on potential mechanical LSDV transmission and to assess whether stable flies and horse flies would be able to transmit LSDV when a shorter period between partial feeding on LSDV viremic cattle followed by further feeding on naïve cattle would be applied.

## Results

### LSDV infection of donor animals

**Figure 1:**
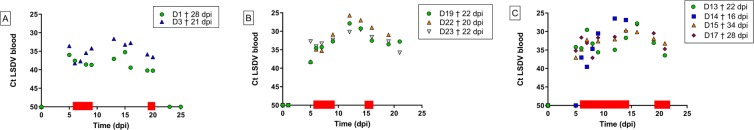
Detection of LSDV DNA by real-time PCR (Ct) in blood donor animals over time (dpi) in Experiment 1 (**A**), Experiment 2 (**B**), Experiment 3 (**C**). Red bars on X-axis indicate the time periods during which *S. calcitrans* flies and *Haematopota spp*. were placed for 10 minutes each day on infected donor animals to obtain a partial infectious blood meal.

**Figure 2:**
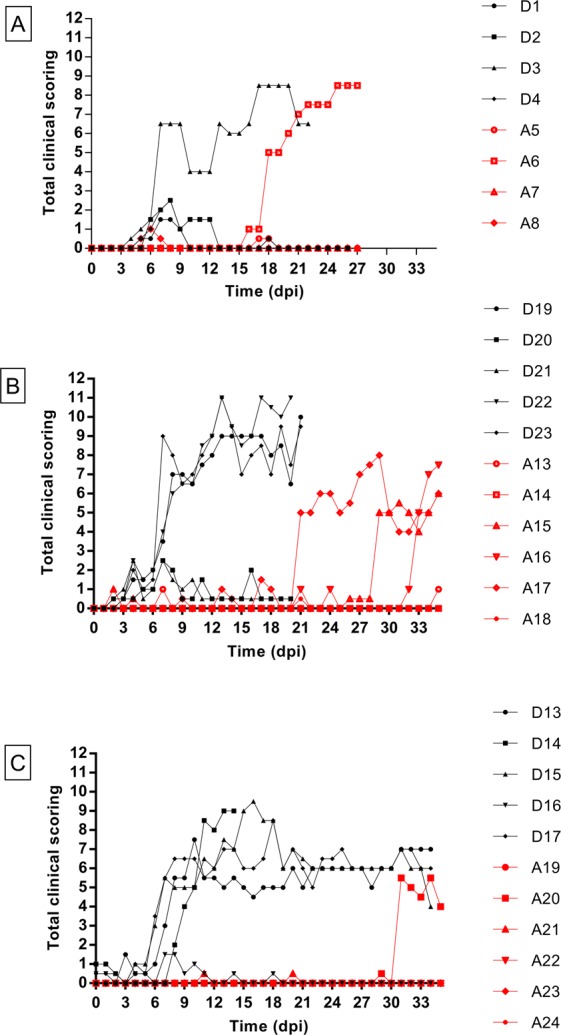
(**A**–**C**) Total clinical scoring XP1 (**A**), XP2 (**B**) and XP3 (**C**) of donor and acceptor animals based on several parameters: fever, food uptake, swelling at inoculation site, number of nodules, location of nodules, erythematous area and lymph node swelling.

All donor animals showed a transient raise in body temperature around 7 dpi. In XP1, 2 (D1, D3) of the 4 donor animals became viraemic from 5 dpi onwards till at least 20 dpi. In XP2, blood samples from 3 (D19, D22, D23) out of 5 donor animals tested positive from 5 till at least 19 dpi. In XP3, 4 out of 5 (D13, D14, D15, D17) donor animals became viraemic from 5 or 6 dpi onwards till 20 dpi. Cycle threshold (C_t_) values varied between 25 and 41 and peak values were between 10 and 16 dpi (Fig. 1). Eight out of 14 (57%) donor animals (D3, D19, D22, D23, D13, D14, D15, D17) developed generalized infections with multiple nodules characteristic of LSD. Skin biopsies of these nodules were LSDV positive by real-time PCR (Table 1). The increase in the clinical scoring for the infected donor animals can be clearly seen in Fig. 2A–C. The incubation period was defined as the time interval between inoculation till the moment animals became viraemic and was rather constant and ranged from 5 to 7 days in these donor animals (Table 1).

**Table 1:** Detection of LSD DNA by real-time PCR in blood and noduli and clinical diagnosis of the donor animals in experiment 1, 2 and 3.

XP	ID animal	First day blood (dpi)	Peak Ct in blood (dpi)	First appearance noduli (dpi)	Ct Noduli (dpi)	Clinical diagnosis
1	D1	5	35.98 (5)	/	no noduli	subclinical LSD
	D2	/	/	/	no noduli	no LSD
	D3	5	31.63 (13)	6	14.94 (21)	generalised LSD
	D4	/	/	/	no nodules	no LSD
2	D19	5	27.88 (12)	7	18.27 (9)	generalised LSD
	D20	/	/	/	no noduli	no LSD
	D21	/	/	/	no noduli	no LSD
	D22	5	25.72 (12)	8	13.78 (9)	generalised LSD
	D23	5	29.57 (14)	7	16.98 (8)	generalised LSD
3	D13	5	27.82 (16)	6	12.83 (8)	generalised LSD
	D14	6	26.88 (14)	8	12.98 (14)	generalised LSD
	D15	5	29.70 (14)	6	14.04 (34)	generalised LSD
	D16	/	/	/	no noduli	no LSD
	D17	5	28.10 (16)	6	15.85 (28)	generalised LSD

XP: experiment. dpi: days post infection.

### LSDV infection of acceptor animals by Stomoxys calcitrans

**Figure 3:**
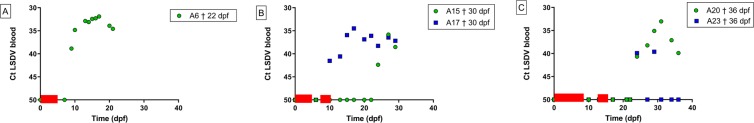
Amount of LSDV DNA (Ct) over time in blood of acceptor animals found positive by real-time PCR in Experiment 1 (**A**), Experiment 2 (**B**) and Experiment 3 (**C**). Red bars on X-axis indicate the time periods during which *S. calcitrans* flies were placed on acceptor animals to continue interrupted feeding and potentially transmit LSDV.

LSDV transmission by *Stomoxys calcitrans* was evidenced in 3 independent experiments (Table 2, Fig. 3). In XP1, only 1 acceptor animal (A6) out of the 4 tested PCR positive from 9 dpf onwards while in XP2 two (A17, A15) of 4 acceptor animals became PCR positive from respectively 10 dpf and 24 dpf onwards. In XP3, 2 (A20, A23) out of 5 acceptor animals were PCR positive. A20 became viraemic from 24 dpf onwards. A23 was only 2 days PCR positive on 24 and 29 dpf. In total, LSDV transmission by *Stomoxys calcitrans* leading to generalized LSD was evidenced in about 30% (4 out of 14) of the acceptor animals (Table 2). The increase in the clinical scoring for the infected acceptor animals can be clearly seen in Fig. 2A–C.

**Table 2:** Summary of results of viraemic acceptor animals after transmission of LSDV by 2 different vectors and incubation period of LSDV.

XP	ID ani-mal	Vector	Incubation period: min-max (days)	First day blood (dpf)	Peak Ct in blood (dpf)	First appearance noduli (dpf)	Ct Noduli (dpf^*^)	First day VNT positive (dpf)	Clinical diagnosis
1	A6	*S. calcitrans*	6–9	9	31.9 (17)	12	11.38 (13)	17	generalised LSD
2	A15	*S. calcitrans*	14–24	24	35.82 (27)	23	30.60 (24)	29	generalised LSD
	A17	*S. calcitrans*	6–10	10	34.49 (17)	15	13.94 (17)	17	generalised LSD
	A16	*Haematopota spp*.	24–26	26	32.57 (27)	26	12.51 (27)	Neg	generalised LSD
3	A20	*S. calcitrans*	12–27	24	33.03 (31)	25	16.61 (28)	31	generalised LSD
	A23	*S. calcitrans*	16–24	24	39.61 (29)	none	none	Neg	subclinical LSD

dpf: days post feeding. VNT: Virus neutralization test.

The development of neutralizing antibodies against LSDV was followed up over time by VNT. Neutralizing antibodies were only detected in the 4 acceptor animals that developed nodules (Table 2) and this between 5 and 8 days after the first detection of LSDV by real-time PCR in blood and coinciding with the peak of viremia. A23 did not develop nodules and had no positive results by VNT, probably due the fact that it only became infected at the end of the experiment.

In the acceptor animals the incubation period, defined as the time between the first time that insects were placed on the acceptor and the first detection of LSDV by real-time PCR in blood, varied between 6 and 27 days (Table 2) but could not exactly be determined since acceptor animals were exposed to multiple batches of flies, as happens in the field, and it is unknown which batch exactly gave rise to virus transmission. A group with a short incubation period (6 to 10 days) (A6, A17) and a group with a long incubation period between 12 to 27 days (A15, A20, A23) can be observed (Table 2).

### LSDV infection of acceptor animals by *Haematopota spp*

**Figure 4:**
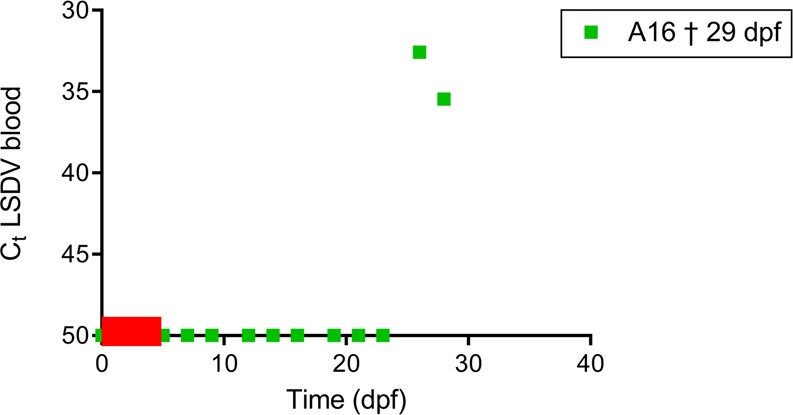
Amount of LSDV DNA (Ct) over time in blood of acceptor animal A16 found positive by real-time PCR in Experiment 2 (B). Red bars on X-axis indicate the time periods during which *Haematopota spp*. horseflies were placed on acceptor animals to continue interrupted feeding and potentially transmit LSDV.

Evidence for LSDV transmission by *Haematopota spp*. was found in XP2. One (A16) out of 2 acceptor animals developed LSD with generalized nodules. Both blood and biopsies of the nodules were PCR positive (Table 2, Fig. 4). No positive results were observed by VNT, probably because this animal was euthanized on 29 dpf, only 3 days after viraemia was detected. The increase in the clinical scoring for A16 can be clearly seen in Fig. 2B. The incubation period here ranged between 24 and 26 days (Table 2).

### Clinical score results and LSDV in tissues and organs at necropsy

**Figure 5:**
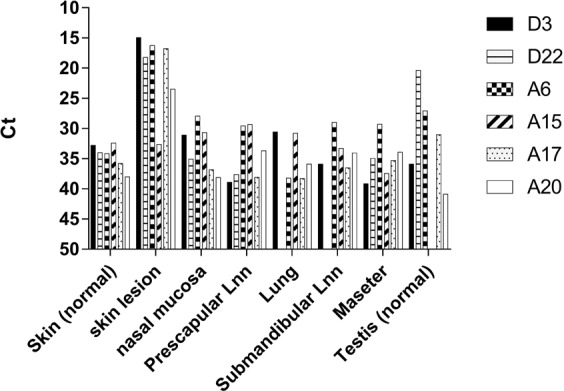
The D5R real-time PCR results from a representative set of tissue samples and organs taken during autopsy from 2 donors (D3, D22) and 4 acceptors (A6, A15, A17, A20). Lnn: Lymph nodes.

The amount of LSDV present in a representative set of tissue samples and organs collected at the end of the experiments from 2 donors (D3, D22) and 4 acceptors (A6, A15, A17, A20) with generalized LSD are presented in Fig. 5 as examples. Between these donor and acceptor animals, visually no obvious difference could be seen during autopsy nor statistically differences (Mann-Whitney test) were found in C_t_ values of the different organs and tissues.

### Detection of LSDV DNA in Stomoxys calcitrans and *Haematopota spp*

**Figure 6:**
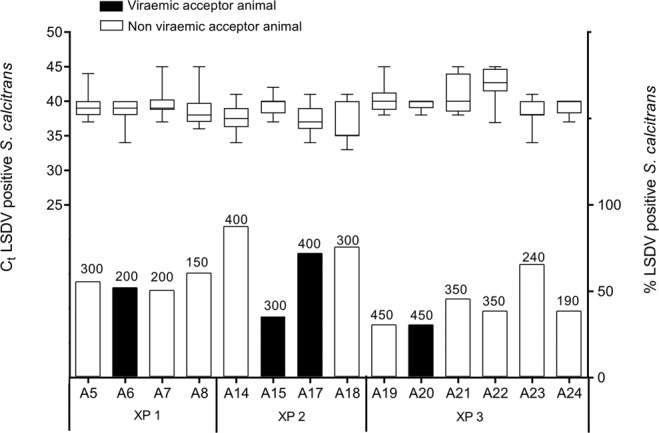
Box plots represent median, 25 to 75 percentiles, minimum and maximum Ct values of LSDV DNA detection in *S. calcitrans* flies and bars represent % LSDV positive *S. calcitrans* flies per acceptor animal.

To investigate whether LSDV infected acceptor animals had been exposed to higher number of infected flies and/or flies carrying a higher viraemic load than uninfected acceptor animals, at least 20 randomly selected *S. calcitrans* flies per acceptor animal were tested in real-time PCR to detect viral DNA. Overall, 52% of all *S. calcitrans* flies tested LSDV positive. No significant difference could be found between the number of infected flies that fed on acceptor animals that became infected (50% infected flies) and on uninfected acceptor animals (53% infected flies) (Mann-Whitney test: p = 0.8). The C_t_-values in all samples were high (mean C_t_ positive *S. calcitrans = *39.14), indicating a low amount of virus (Fig. 6). No significant difference in mean C_t_ values of the flies were found between the group of viraemic and non viraemic acceptor animals (Mann-Whitney test: p = 0.4).

**Figure 7:**
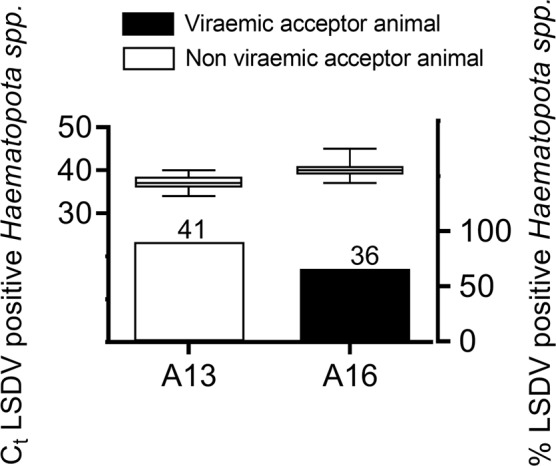
Box plots represent median, 25 to 75 percentiles, minimum and maximum Ct values of LSDV DNA detection in *Haematopota spp*. and bars represent % LSDV positive *Haematopota spp*. per acceptor animal.

A similar analysis was performed on the horseflies. Also here, no relationship between the number of LSDV positive horseflies and LSDV transmission could be found since the uninfected acceptor animal was exposed to more LSDV positive horseflies (90%) than the acceptor that became LSDV infected (66%). The overall mean C_t_ of positive *Haematopota spp*. was 38.85 (Fig. 7). No significant difference in mean C_t_ values of the horseflies were found between the viraemic and non viraemic acceptor animals (Mann-Whitney test: p = 0.4).

## Discussion

With this study, we provide the first evidence of LSDV transmission by *Stomoxys calcitrans* and *Haematopota spp*. Although epidemiological observations already suggested the role of stomoxys flies in the transmission of LSDV^20^, this is the first formal demonstration under experimental conditions of *S. calcitrans* as vector of LSDV. The fact that LSDV was transferred from donor to acceptor animals by flies that had been exposed to the virus for a maximum of 3 days, and for animal A23 even after 1 day, provides strong indications that this transmission was mechanical, and not biological. This is in line with other work^8^ that indicates no evidence of viral replication in the insect vector and also suggests that the mode of transmission must be mechanical and not biological.

It is known that not all animals exposed to LSDV develop clinical infection. About half of experimentally infected animals exhibit clinical signs^22^ and the morbidity rate after natural infection varied between 2.6 and 42% in the period 2015–2017 in the Balkan countries^23^. This is in line with our results since 57% of our donor animals, that received a high viral dose via artificial inoculation, progressed to clinical LSD, as well as 30% of the acceptor animals which were inoculated via our stomoxys flies.

At this moment, we cannot irrefutably explain why some acceptor animals become LSDV infected and others not while they were all exposed to a similar number of LSDV infected flies carrying comparable viral loads. A potential explanation could be found in the genetic diversity between individual animals or in differences in their immune status at the moment of infection. The experiments were namely performed with cattle purchased from regular farms and not with ‘specific pathogen free’ animals.

Another shortcoming of our current experimental setup is that we cannot determine how many LSDV infected flies continued their blood meal on the acceptor animals and thereby transmitted the virus. In the absence of this information, an acceptable hypothesis could be that some acceptor animals were bitten by more infected flies than others and thereby received a higher viral dose that passed the minimal infectious dose leading to clinical LSD. It seems namely probable that one stable fly cannot transmit sufficient virus to induce LSD. Stomoxys flies are described to carry only 0.4 nl^24^ of blood on their mouth parts what fits with the low levels of LSDV DNA we detected in real-time PCR. So even when there is 10^5.6^ TCID50/ml of virus present in the blood at the peak of viremia^25^, only 10^−0.8^ TCID50 are transferred per fly. This seems a very low dose, although no actual data is present in literature on the dose necessary to start an infection. Another option that cannot be excluded is that a higher viral dose is inoculated by regurgitation from the crop during blood feeding^26^. However, stable flies have only been shown to regurgitate under artificial conditions, but no convincing studies support this as a natural means of pathogen transmission^27^.

In a theoretical approach, we calculated the maximum dose to which acceptor animal A6 that developed LSDV was exposed by stable flies via the following formula: viral dose at peak viremia * volume transmitted by fly * number of flies per animal * number of days of successive feeding * 50% fly infection rate. This led to a maximum inoculated viral dose for A6 of 10^1.4^ TCID50. When compared with the dose of 10 ^8.4^ TCID50 artificially injected per donor animal, it indicates that transmission by *S. calcitrans* flies is rather efficient and that saliva components might enhance LSDV infection compared to artificial needle inoculation.

Furthermore, our results show that horseflies also transmit LSDV and even might do this more efficiently than stable flies since less horseflies were put on the acceptor animals and 1 out of 2 became positive. The large mouthparts of tabanids lend themselves well to mechanical transmission as they can retain a high blood volume, and thus inoculate higher viral doses during interrupted feeding between hosts. The blood volume retained in the tabanids’ mouthparts (*Tabanus fuscicostatus****)*** was evaluated at 12.5 nl^28^, which is much larger than the volume of blood retained in the mouthparts of *S.calcitrans* (0.4 nl)^24^. Although the interval between complete blood meals for horseflies is quite long (5–7 days), probably resulting in inactivation of most pathogens on the mouthparts^29^, the bites are very painful and lead to a need for multiple bites during a short time period to obtain complete blood meal^30^ and create thereby multiple occasions for viral transmission. Taking into account that tabanids are highly seasonal in temperate regions^31^ and only females feed on blood, horseflies probably play a less important role in field conditions than the abundant stable flies which feed every 4 to 72 hours^16^.

Interestingly, once viremia was detected both in artificially and vector inoculated animals, a similar pathogenesis, immune response and disease progression was observed irrespective of the inoculation route or dose. Nevertheless, an important difference in the incubation period was apparent between artificially inoculated donor animals and naturally inoculated acceptor animals by the vector. The incubation period in donors was rather stable (5–7 days), while this ranged between 6 and 26 days in acceptors, although we cannot pinpoint the exact moment and dose that was inoculated in the acceptor animals due to the experimental set-up. This variation in the length of the incubation period in acceptors is probably also related to the inoculated viral dose. Future studies should be conducted to study this in more detail since the duration of the incubation period is crucial to make decisions on control measures and to guarantee the effectiveness of quarantine zones.

Our findings on mechanical transmission of LSDV by *S. calcitrans* and *Haematopota spp*., may have important implications for the control measures against LSD. Animal movement restriction should be implemented during active vector periods and infected animals should be taken out (eradication) or should be separated from susceptible cattle. This separation or quarantaine should last sufficiently long, seen our finding that the incubation period could take up to 27 days and maybe longer. Besides the use of appropriate vaccines in controlling LSD^32,33^, it should also be considered to implement an integrated vector management approach^20,34^.

## Methods

### Virus isolates

The LSDV field isolate (LSD/OA3-Ts. MORAN. M. seed pass.4. 155920/2012 .20.1.13) used in this study was recovered from cattle during an outbreak in Israel in 2012. The isolate was grown in lamb testis cell cultures. The infection dose was 10^7.55^ TCID50/ml.

### Insects

**Figure 8:**
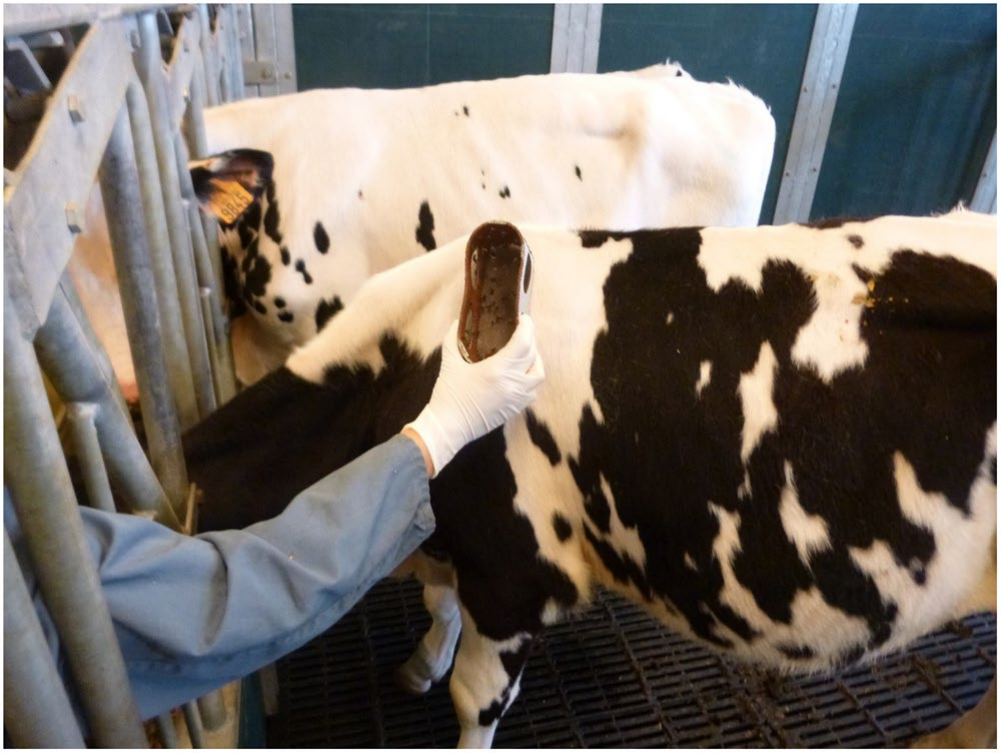
Mode of feeding the insects on donor and acceptor animals.

Biting flies and Tabanids were used in this study since these are considered important vectors for mechanical disease transmission^16^. We more specifically collected *S. calcitrans* and *Haematopota spp*. as these are the most abundant biting flies and Tabanidae, respectively, present in Belgium^17^. Stable flies were caught in the vicinity of a cattle herd (Drongen, Belgium) using insect nets. Horseflies were collected outdoors with H-traps^17^ at an equestrian center (Drongen, Belgium). These insects were subsequently identified using morphological keys^35,36^. Batches of 50–200 *S. calcitrans* and 35–41 *Haematopota spp*. were kept within plastic tubes covered at each end with a mosquito net sleeve for an average of 2 days before the experiment (Fig. 8). During this period, they only received a cotton pledge moisted with water.

### Experimental set-up

#### General

Belgian bulls (4–6 months old, black and white dairy cattle) were used for all XPs. All animals were collected in Belgium which is historical free of LSD. Nevertheless all animals were checked by PCR and by serological tests (VNT) to verify their negative status before the experiments started. Cattle were allowed to acclimatize for 7 days before onset of the trial. Donor and acceptor animals were kept separately in insect-free Biosafety Level 3 facilities (Sciensano, Machelen, Belgium). Water was available ad libitum and animals were fed once each day. The animals were randomly assigned to the donor or acceptor groups. Animal experiments were performed in accordance with the European Union and Belgian regulations on animal welfare in experimentation. The protocol was approved by the joined ethical committee of Sciensano, authorisation number 20150605-01.

#### Experimental infection of donor animals

In analogy with the challenge method used for the LSD vaccine efficacy test^37^, the donor animals (Table 1) were inoculated with virus stock intravenously (6 ml) in the vena jugularis and intradermal (1 ml). The latter was done by injecting 250 µl in 4 different locations on both sides of the neck.

#### Virus transmission to acceptor animals by *S. calcitrans* and *Haematopota spp*

Three independent experiments (XP) were performed wherein insects were allowed to feed for 10 minutes on LSDV infected bulls at the moment of viremia or when nodules began to appear, since the highest virus load is found in the skin lesions^25^. Transmission of virus was then attempted by allowing potentially infected insects to feed for ten minutes on susceptible cattle at one hour post-infective feeding (Fig. 8). The exact number of insects and the moment of attempted transmission is shown in Table 3. The first day of feeding of the insects on the acceptor animals was designated as day 0 post feeding (0 dpf). Transmission was confirmed by recording clinical signs of LSD and virus detection in blood and noduli of acceptor animals.

**Table 3:** Set-up in experiment 1, 2 and 3 with the exact number of insects and the moment of attempted transmission from donor to acceptor animals.

XP	Vector	#	Dpi	Donor animals					Acceptor animals					
**1**				**D1**	**D2**	**D3**	**D4**		**A5**	**A6**	**A7**	**A8**		
	S.calcitrans	400	6	200						100		100		
			6			200			100		100			
			7			400			100	100	100	100		
	*S.calcitrans*	250	7–8–9			250			100	100		50		
	*S.calcitrans*	200	19–20			200			100		100			
**2**				**D19**	**D20**	**D21**	**D22**	**D23**	**A14**	**A15**	**A17**	**A18**	**A13**	**A16**
	*S.calcitrans*	800	6–7–8	200								200		
			6–7–8				400		200		200			
			6–7–8					200		200				
	*S.calcitrans*	200	7–8–9				200		100		100			
	*Haematopta spp*.	36	7–8–9	36										36
	*Haematopta spp*.	41	7–8–9					41					41	
	*S.calcitrans*	400	15–16	100					100					
			15–16				100			100				
			15–16					200			100	100		
**3**				**D13**	**D14**	**D15**	**D16**	**D17**	**A19**	**A20**	**A21**	**A22**	**A23**	**A24**
	*S.calcitrans*	400	6	200					100	100				
			6			200					100	100		
	*S.calcitrans*	400	7	100					100					
			7		200					100	100			
			7			100						100		
	*S.calcitrans*	400	8	100					100					
			8		100					100				
			8			100					100			
			8					100				100		
	*S.calcitrans*	340	9	150					50	50		50		
			9			50					50			
			9				140						70	70
	*S.calcitrans*	100	12	50									50	
			12			50								50
	*S.calcitrans*	50	13			50							50	
	*S.calcitrans*	240	14			120								120
			14					120					120	
	*S.calcitrans*	200	19–20–21	100					100					
			19–20–21			100				100				

XP: experiment. ^#^exact number of insects. dpi: days post infection.

In XP1 and 2, insects were placed on the animals for 2 to 3 consecutive days. In XP3, batches of *S. calcitrans* were only allowed to feed once on donor animals and subsequently on acceptor animals from 6 to 14 days post infection (dpi). From 19 to 21 dpi, transmission of LSD virus was attempted with feeding for 3 consecutive days for acceptors A19 & A20. All insects were afterwards put in the −20 °C freezer.

### Monitoring of clinical signs in animals

Several parameters were collected throughout the duration of the trial: fever, food uptake, swelling at inoculation site, number of nodules, location of nodules, erythematous area and lymph node swelling and each parameter got a score between 0–2. Clinical diagnosis was divided in generalized (generalized nodules, spread to organs, positive real-time PCR), mild (only local nodules, positive real-time PCR), subclinical (no nodules, positive real-time PCR) and no LSD (no symptoms, negative real-time PCR). The day of virus inoculation was designated as 0 dpi. The experiments lasted 42 days after inoculation of donor animals, followed by euthanasia and subsequent necropsy. Some animals were euthanized earlier for welfare reasons.

### Sample collection

EDTA blood samples and biopsies of nodules were collected for real-time PCR analysis. Serum samples were collected for virus neutralization test (VNT). At the end of the experiments, tissues and organs were collected for real-time PCR (Fig. 5).

### Real-time PCR

DNA was extracted from blood, tissue and organ samples using the Nucleo Spin Blood and Nucleo Spin tissue kits. For DNA extraction from insects, these were first individually homogenized in 1 ml T1 buffer and proteinase K using beads in a Tissue Lyser (6 min, 250 Hz) (Qiagen, Gaithersburg, MD, USA) followed by overnight incubation at 56 °C. DNA was then extracted using the Nucleo Spin tissue kit^38^. The D5R PCR^38^ was used to check a minimum of 20 randomly selected *S. calcitrans* and all *Haematopota spp*. for each acceptor animal.

The detection of capripox viral genome was carried out using the real-time PCR panel as described by Haegeman *et al*.^38^ All samples were first screened with the D5R real-time PCR. Samples with Ct-values above 37 were confirmed with the E3L and J6R real-time PCRs. A sample was considered to be positive if: 1) the Ct < 37 or 2) when at least 2 out of the 3 real-time PCR’s had a Ct-value. For blood and tissue samples, the internal and external control Ct-values were analysed and followed in order to reduce the impact of sample and extraction quality variability. For insects, this was only the external control. Control samples were added during the extraction (negative control) and PCR setup (2 positive and 1 negative control). A standard curve for the D5R real-time PCR (Supplementary Fig. S1) can be used to determine copy numbers.

### Virus neutralization test

The virus neutralization test was performed on OA3.T cells (ATCC-CRL-6546, LGC standards, Middlesex, United Kingdom) grown to confluency in 96 well plates according to the OIE Terrestrial Manual^37^, except for the visualization of the non-neutralized virus which was carried out as described in Haegeman *et al*.^39^.

### Statistics

All statistical analyses (Mann-Whitney U tests) were performed using SPSS statistics software version 25 (IBM). P values < 0.05 were considered to be significant.

## Supplementary information

Supplementary Figure S1
