# Supplementary material for: Experimental evidence of mechanical lumpy skin disease virus transmission by Stomoxys calcitrans biting flies and Haematopota spp. horseflies
Source: Sci Rep. 2019 Dec 27;9:20076. doi: 10.1038/s41598-019-56605-6 (PMC6934832; doi:10.1038/s41598-019-56605-6)
Supplement: Supplementary file 1 — Supplementary Figure S1 [file 41598_2019_56605_MOESM1_ESM.pdf]

Experimental evidence of mechanical lumpy skin disease virus transmission by *Stomoxys calcitrans* biting flies and *Haematopota spp.* horseflies

C. Sohier*#1, A. Haegeman# 1, L. Mostin 1, I. De Leeuw[1], W. Van Campe[1], A. De Vleeschauwer[1], E.S.M. Tuppurainen[2], T. van den Berg[1], N. De Regge[1], K. De Clercq[1]

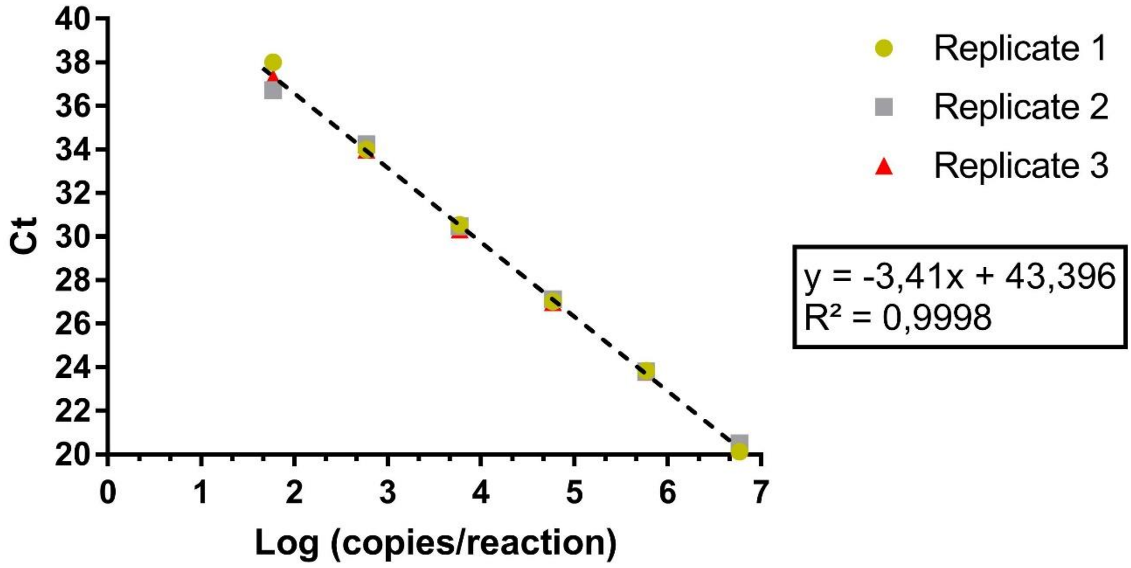

Supplementary Figure S1: Standard curve for the D5R real-time PCR with Ct values and log/copies numbers
